# Prognostic models for the early care of trauma patients: a systematic review

**DOI:** 10.1186/1757-7241-19-17

**Published:** 2011-03-20

**Authors:** Marius Rehn, Pablo Perel, Karen Blackhall, Hans Morten Lossius

**Affiliations:** 1 Department of Research, Norwegian Air Ambulance Foundation, Drøbak, Norway; 2 Akershus University Hospital, Lørenskog, Norway; 3 University of Oslo, Faculty Division Oslo University Hospital, Kirkeveien, Oslo, Norway; 4 Nutrition and Public Health Intervention Research Unit, Epidemiology and Population Health Department, London School of Hygiene & Tropical Medicine, London, UK; 5 Department of Surgical Sciences, University of Bergen, Bergen, Norway

## Abstract

**Background:**

Early identification of major trauma may contribute to timely emergency care and rapid transport to an appropriate health-care facility. Several prognostic trauma models have been developed to improve early clinical decision-making.

**Methods:**

We systematically reviewed models for the early care of trauma patients that included 2 or more predictors obtained from the evaluation of an adult trauma victim, investigated their quality and described their characteristics.

**Results:**

We screened 4 939 records for eligibility and included 5 studies that derivate 5 prognostic models and 9 studies that validate one or more of these models in external populations. All prognostic models intended to change clinical practice, but none were tested in a randomised clinical trial. The variables and outcomes were valid, but only one model was derived in a low-income population. Systolic blood pressure and level of consciousness were applied as predictors in all models.

**Conclusions:**

The general impression is that the models perform well in predicting survival. However, there are many areas for improvement, including model development, handling of missing data, analysis of continuous measures, impact and practicality analysis.

## Background

Trauma is a major global contributor to premature death and disability. The burden of injuries is especially notable in low and middle-income countries and is expected to rise during the coming decades [1,2]. Harm from major trauma may be minimized through early access to pre-hospital [2] and in-hospital trauma care [3]. A majority of trauma related deaths occur during the pre-hospital period or in the initial hours after injury. Emergency medical service (EMS) providers must therefore rapidly assess trauma severity in order to identify patients that require prompt referral to an appropriate hospital [2,3] and to ensure that necessary diagnostic and therapeutic interventions are initiated upon admission. However, early recognition of major trauma remains a challenge due to occult injuries, unpredictable evolution of symptoms, and the complexities of evaluating patients in the early hours after injury.

If patients only suffering minor injuries bypass the local clinic (overtriage; false-positives), the regional hospital will be overwhelmed and create a strain on scarce financial and human resources. However, if major trauma victims are treated at the local clinic rather than being stabilized and rapidly transported to a facility providing higher level of trauma care (undertriage; false-negatives), avoidable deaths may occur. Sensitivity and specificity are often negatively correlated making optimal prognostic model performance a balance between patient safety and optimal resource utilisation. American College of Surgeons-Committee on Trauma (ACS-COT) therefore describes 5% undertriage as acceptable and associated with an overtriage rate of 25% - 50% [4].

At hospital admission, delay to high resource resuscitation can result in unfavourable outcome [5,6]. Traditionally, these early decisions have been informed by the patient's injury severity. In this context, severity has been defined by the patient's risk or prognosis. Although commonly used interchangeably, risk and prognosis differ in their meaning. Prognosis can be defined as "*the probable course and outcome of a health condition over time*" [7]. Risk is sometimes used as a synonym of probability, but it can also used as a synonym for hazard [8]. We believe the term prognosis is more appropriate in this context and will use this term throughout this manuscript.

Assessment of injury severity traditionally includes clinical findings pertaining to physiological derangement, obvious anatomical injury, mechanism of injury, and pre-injury health status. These individual variables have been useful to predict a patient's prognosis in trauma (i.e. predictors), but have showed limitations when used as isolated parameters [9].

To overcome the limitation of individual characteristics, different predictors can be combined into scores or models to estimate patient's prognosis and guide EMS providers in their early evaluations of these patients. Prognostic models in the context of trauma are also referred to as risk models, prognostic scores, triage scores or risk scores. The abundances of prognostic models in the trauma setting indicate not only the need for early objective quantification of prognosis, but also the difficulties of addressing all requirements to be valid, precise and practical.

The ideal prognostic model for trauma should be developed following methodological guidelines, it should be clinically sensible, well calibrated and with good discriminative ability [10,11]. Further, it is cost-effective, externally validated, field-friendly and it provides useful information to EMS providers that improve triage decision-making and patient outcome [12-15]. We aim to conduct a systematic review that identifies existing prognostic models aimed at improving early trauma care, appraise their quality and describe their characteristics and performance in order to inform clinical practice and future research.

## Methods

### Study eligibility criteria

We included studies reporting prognostic trauma models that were developed to improve clinical decision-making in the field and upon immediate arrival to hospital.

We defined "prognostic model" as a tool for clinicians that includes 2 or more predictors obtained from the history and physical examination of a suspected trauma victim (Glasgow Coma Scale (GCS) [16] was considered to be a single predictor). Because we were interested in the models that could be used early in the assessment of trauma patients, we only included models with predictors collected in the field or in the emergency department up to 12 hours from injury. Further, we did not include models that required complex information such as para-clinical diagnostic tests (e.g. blood sampling) or models for organ specific injuries. Studies that investigated more than one predictor but did not combine them in a model (e.g. field triage decision schemes) were also excluded. We included studies that aimed to derivate prognostic models (derivation studies) or validate them (validation studies).

We included only prognostic models developed for adult patients defined, for the purpose of this review as over 15 years of age or if the patients were described by the authors as adults. This is due to differences between paediatric and adult physiology. Studies that aimed to derivate a prognostic model pertaining to adult trauma patients, but failed to report population age were included.

Models pertaining to burns, drowning, strangulation, isolated proximal femur fractures, isolated traumatic brain injury, pregnancy or medical conditions were excluded. We only included studies within the last 20 years. Studies conducted prior to 1989 were excluded because patient management and diagnostic techniques have changed considerably since then. Studies published in the inclusion period that validated prognostic models developed in the period 1982-89 were included and the original derivation study was assessed. Studies not written in English were excluded. The review was conducted according to PRISMA guidelines [17]. Being a systematic literature review, this study did not need approval from The Regional Committee for Research Ethics.

### Study identification, selection and data extraction

A systematic literature search of MEDLINE to identify relevant studies was conducted (KB) (see additional file 1 for search strategy). All studies were collated in an Endnote bibliographic database (^© ^2007 Thomson Reuters). Two reviewers (MR & PP) independently examined titles, abstracts and keywords for eligibility. The full texts of all potentially relevant studies were obtained and two reviewers (MR & PP) assessed each study using pre-defined inclusion criteria (see additional file 2 for excluded full text studies with reasons). The bibliographies of all included studies were inspected for further relevant studies. Two reviewers (MR & PP) used a customized Excel spreadsheet (^© ^2007 Microsoft Corporation) to record extracted information from the selected studies in order to examine study characteristics and to appraise methodological quality.

### Study characteristics

From all included studies, we collected descriptive data on study population and economic region (high income, middle income and low income countries). We also depicted study objective (derivation or validation study) as well as predictors. Finally, we described relevant study outcomes (mortality, morbidity or process outcomes), anatomic injury and measures of accuracy.

### Quality appraisal of prognostic models

Assessment of methodological quality was facilitated through the application of a 17-item long quality appraisal list (see additional file 3). The list focussed on two areas:

a) Internal validity (to what extent is systematic error (bias) minimized).

b) External validity (to what extent can the prognostic model correctly be applied to other populations).

The internal validity and some items from the external validity (items 1 to 14) were only assessed in the original study that derived the prognostic model (derivation studies).

Depending on study design, some quality items are more relevant than others. It therefore proved difficult to determine the weight that each item should contribute to the overall score. We avoided the use of a quality assessment score; as such scores are debated [18,19]. Instead we described key components of methodological quality separately.

### Performance of prognostic models externally validated

We collected performance data and focused on sensitivity/specificity, receiver operating characteristic (ROC) or area under ROC curve (AUC), when several measures of accuracy were portrayed. We focused on survival when several outcome measures were reported.

## Results

### Literature search

We identified 4 880 records from the MEDLINE search (see additional file 1 for the MEDLINE search strategy) and added additional 59 records identified through reference lists of selected studies identified in the initial search. We screened a total of 4 939 records of which 143 were assessed in full text for eligibility.

We included 5 studies [20-24] that derived 5 prognostic models and 9 studies [25-33] that validated one or more of these models in external populations.

**Figure 1:**
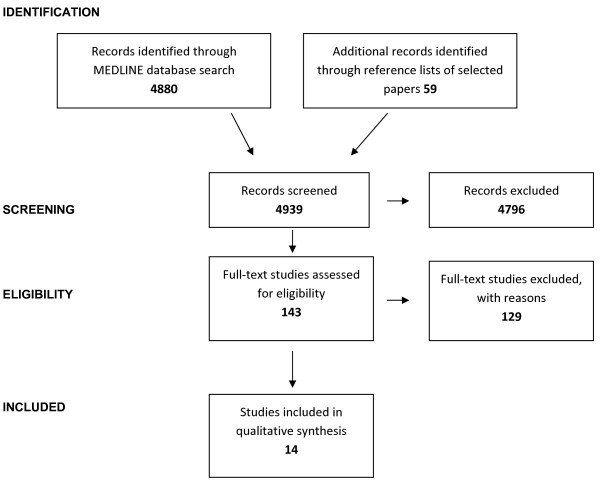
**Information flow through the different phases of the systematic review**.

Among the 129 full text studies excluded with reason, 7 validation studies were found ineligible as they included children (see additional file 2). Figure 1 shows a PRISMA diagram [17] to depict the flow of information through the different phases of the systematic review.

### Characteristics and performance of the prognostic models

Table 1 depicts the prognostic models with their corresponding predictors and scoring systems. Systolic blood pressure and level of consciousness were considered predictors in all models.

**Table 1:** Presentation of prognostic models included in the review

CRAMS		PHI		T-RTS		PSS		MGAP	
**Circulation**		**SBP**		**SBP**		**SBP**		**SBP**	
normal CR and SBP > 100	2	>100	0	>89	4	>90	4	>120	5
delayed CR or SBP 85-100	1	86-100	1	76-89	3	70-90	3	60-120	3
no CR or SBP < 85	0	75-85	2	50-75	2	50-69	2	<60	0
**Respiration**		0-74	5	1-49	1	<50	1	**MOI**	
normal	2	**Pulse**		no pulse	0	no pulse	0	Blunt	4
abnormal	1	≥120	3	**Respiration (RR)**		**Respiration (RR)**		**Age**	
absent	0	51-119	0	10-29	4	10-24	4	>60	5
**Abdomen/thorax**		<50	5	>29	3	25-35	3	**Consciousness**	
nontender	2	**Respiration**		6-9	2	>35	2	GCS	*)
tender	1	normal	0	1-5	1	1-9	1		
rigid/flail chest	0	labored/shallow	3	0	0	0	0		
**Motor**		RR < 10/needs intubation	5	**GCS**		**Consciousness**			
normal	2	**Consciousness**		13-15	4	normal	4		
resonse to pain	1	normal	0	9-12	3	confused	3		
no response	0	confused	3	6-8	2	responds to sound	2		
**Speech**		no intelligible words	5	4-5	1	respons to pain	1		
normal	2			3	0	no response	0		
confused	1								
no intelligible words	0								

**Score range**									
0-10		0-20		0-12		0-12		3-29	

**Note**: CRAMS = Circulation, Respiration, Abdomen, Motor, Speech; PHI = Pre-Hospital Index; T-RTS = Triage-Revised Trauma Score; PSS = Physiologic Severity Score; MGAP = Mechanism, Glasgow Coma Scale, Age, and Arterial Pressure; CR = Capillary Refill; SBP = Systolic Blood Pressure; GCS = Glasgow Consciousness Scale; MOI = Mechanism of Injury; RR = Respiratory Rate; *) GCS value

#### Circulation, Respiration, Abdomen, Motor, Speech (CRAMS)

The CRAMS was derived on 500 North American patients by Gormican in 1982 [20]. The derivation study included consecutive paramedic runs involving trauma and collected predictors both in the pre-hospital and early in-hospital phase. The CRAMS utilise predictors pertaining to capillary refill, systolic blood pressure (SBP), respiration, tenderness of the abdomen or thorax, motor response and ability to speech. The model predicts outcomes pertaining to need for emergency general- or neurosurgery and emergency department (ED) mortality.

#### Pre Hospital Index (PHI)

The PHI was derived on 313 North American patients by Koehler et al. in 1986 [21]. They included consecutive trauma patients to identify relevant model predictors easily obtained in the pre-hospital phase. Numerical weight assignments were performed on the same 313 patients. The PHI includes variables pertaining to SBP, heart rate, respiration and level of consciousness to predict the need for emergency general- or neurosurgery and 72 hours post injury mortality.

#### Triage Revised Trauma Score (T-RTS)

Champion et al. developed the Revised Trauma Score (RTS) and the Triage-Revised Trauma Score (T-RTS) in 1989 [22] as a revision of the Trauma Score [34]. The T-RTS is used in the clinical context for triage and clinical decision-making, whereas the RTS is used by researchers and administrators for case mix control and benchmarking.

The RTS was developed using the MTOS database (over 26 000 subjects), but the exact number of patients included in the development is unclear. The RTS uses the weight given by the logistic regression analysis and provides an outcome prediction. The weighted RTS ranges from 0 to 7,84 and is not considered to be a prognostic model for the early care of trauma patients in this review.

The T-RTS was derived on admission physiology data on 2 166 North American consecutive trauma patients included in a trauma centre database. Champion et al. divided SBP and respiratory rate (RR) into integers that approximated the intervals chosen for GCS. The T-RTS varies from 0-12 and predicts Injury Severity Score [35] (ISS) > 15 and survival at end of acute care/hospital discharge. The T-RTS is simple to use and is included as a prognostic model in this review.

#### Physiologic Severity Score (PSS)

The PSS by Husum et al. was derived in 2003 on 717 patients injured in North Iraq and Northwest Cambodia [23] as a simplification of the T-RTS [22]. They collected pre-hospital data on consecutive trauma patients and included predictors pertaining to SBP, RR and level of consciousness. The model predicts survival during pre-hospital evacuation and hospital stay as well as ISS > 14.

#### Mechanism, Glasgow Coma Scale, Age, and Arterial Pressure (MGAP)

The MGAP was derived on 1 360 French patients by Sartorius et al. in 2010 [24]. They included pre-hospitally collected data on consecutive trauma patients to identify relevant model predictors. The MGAP utilise SBP, mechanism of injury, age and GCS to predict 30-day mortality.

All the prognostic models utilized different times of survival as the primary endpoint. Two studies [20,21] included the need for emergency general or neurosurgery, whereas ISS was evaluated as an outcome in two studies [22,23].

Table 2 describes performance in the derivation and validation samples. There was clinically significant heterogeneity in the performance of the same prognostic model in different validation studies. Additional file 4 depicts characteristics of investigated outcomes.

**Table 2:** Performance of prognostic models

Model Derivation study (No. pts; Country)	Study (No.pts; Country)	Main outcome	Performance
**CRAMS**	**Gormican-82**∞ (500 pts; USA)	Survival or emergency surgery	CRAMS < 9: Sens = 92%; Spec = NA
	
	Baxt-89 (2 434 pts; USA)	Survival	ROC-curves presented, AUC = NA
	
	Emerman-92 (1 027 pts; USA)*	Survival	CRAMS < 9: Sens = 100%; Spec = 83%

**PHI**	**Koehler-86 ∞ (**465 pts; USA)	Survival or emergency surgery	PHI > 3 = Sens = NA; Spec = NA
	
	Koehler-86 (388 pts; USA)	Survival or emergency surgery	PHI > 3: Sens = 94,4%; Spec = 94,6%
	
	Baxt-89 (2 434 pts; USA)	Survival	ROC-curves presented, AUC = NA
	
	Emerman-92 (1 027 pts; USA)	Survival	PHI > 3: Sens = 100%; Spec = 88%
	
	Plant-95 (621 pts; Canada)	Survival	PHI > 3: Sens = 98%; Spec = 54%
	
	Bond-97 (3147 pts; Canada)	ISS > 15	PHI > 3: Sens = 41%; Spec = 98%
	
	Tamim-02 (1 291 pts; Canada)	Survival or emergency surgery or ICU admittance	AUC = 0,66

**T-RTS**	**Champion-89 ∞ (**2 166 pts; USA)	ISS > 15	T-RTS < 12: Sens = 59%; Spec = 82%
	
	Baxt-89 (2 434 pts; USA)	Survival	ROC-curves presented, AUC = NA
	
	Emerman-92 (1 027 pts; USA)	Survival	T-RTS < 12: Sens = 100%; Spec = 88%
	
	Roorda-96 (398 pts; The Netherlands)	Survival or emergency surgery or ICU admittance	T-RTS < 12: Sens = 76%; Spec = 94%
	
	Al-Salamah-04 (795 pts; Canada)	Survival	AUC = 0,83
	
	Ahmad-04 (30 pts; Pakistan)	Survival	Mortality = T-RTS 6-7 = 60%, T-RTS 8-10 = 12,5%, T-RTS 11-12 = 8,3%
	
	Moore-06 (22 388 pts; Canada)	Survival	AUC = 0,84
	
	Sartorius-10 (1 003 pts; France)	Survival	AUC = 0,88

**PSS**	**Husum-03**∞(717 pts; Iraq and Cambodia)	Survival	AUC = 0,93

**MGAP**	**Sartorius-10**∞(1 360 pts; France)	Survival	AUC = 0.90
	
	Sartorius-10 (1 003 pts; France)	Survival	AUC = 0,91

∞) Derivation sample; *) Modified CRAMS scale; pts = patients; ROC = Receiver Operating Characteristic; AUC = Area under receiver operating characteristic curve; NA = Not Available; Sens = Sensitvity; Spec = Specificity; CRAMS = Circulation, Respiration, Abdomen, Motor, Speech; PHI = Pre-Hospital Index; T-RTS = Triage-Revised Trauma Score; ISS = Injury Severity Score; PSS = Physiologic Severity Score; MGAP = Mechanism, Glasgow Coma Scale, Age, and Arterial Pressure

### Quality of prognostic models

**Figure 2:**
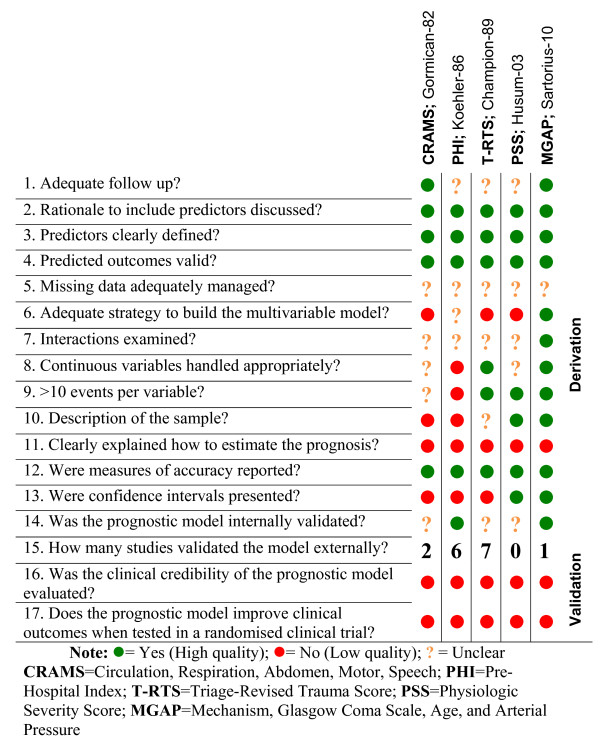
**Quality assessment of prognostic models: Review authors' judgments about each methodological quality item**.

Figure 2 shows the methodological quality items for each included prognostic model.

All derivation studies for the 5 prognostic models discussed the rationale to include the predictors and provided clear definitions. All outcomes seemed valid, but none were clear in their handling of missing data. Examination of interactions and handling of continuous variables were often unclear. None of the studies reported exploration of more complex relationships for continuous variables (e.g. fraction polynomial or spline functions). The only model that was developed using an appropriate multivariable approach was the MGAP. The CRAMS study neither described the process of predictor identification nor the numerical weight assignments. The PSS and the T-RTS aimed to simplify existing models and modified predictors previously presented. The PHI and MGAP models clearly portrayed the internal validation process. However, it remains unclear how the CRAMS, T-RTS and PSS were internally validated.

The CRAMS was externally validated in 2 studies [25,26], the PHI in 6 studies [21,25,26,31-33], the T-RTS in 7 studies [24-30]. The PSS remains unvalidated in an external population, whereas external validation of the MGAP was reported in the derivation study. None of the models clearly explain how to estimate prognosis for individual patients.

In all the original articles (derivation studies) the authors implied that the prognostic models would be useful to change clinical practice, but the clinical credibility of the model remained unevaluated, and none of the models were tested in a randomised clinical trial.

## Discussion

This systematic review located 5 prognostic models for the early care of trauma patients. The majority of models were developed in cohorts of trauma patients from the 80's. All except one of the models were developed in populations from high-income countries. The number of predictors included in the models ranged from three to five, and SBP was the only predictor included in all models. GCS has proven to predict the need for trauma centre admittance [36], but have been criticized for being difficult to score correctly [37,38]. Reflecting this, variously defined predictors depicting consciousness were included in all models. All the prognostic models evaluated survival as an outcome, although the timing was defined differently for all the models. Further, we revealed heterogeneity in outcomes other than survival highlighting the consensus among researchers regarding a common definition of "major trauma" is needed (see additional file 4; Characteristics of investigated outcomes").

All the models, except PSS, were validated in external populations. The T-RTS was the most frequently validated (7 studies). The performance of the prognostic models showed a large variation between different validation studies (see table 2), although the majority of studies were conducted on populations from USA and Canada. The reason for these differences can be related to methodological issues, such as different variable definitions or alternatively it could be related to the difficulty of transporting prognostic model to different settings. Factors that may affect the transportability of prognostic factors could be related with injury characteristics (e.g. penetrating injuries), patient's characteristics (e.g. age), or medical services characteristics (e.g. pre-hospital transportation distances or level of EMS personnel competence).

Importantly, although 80% of trauma deaths occur in low and middle-income countries where many of these characteristics are likely to be different from developed countries, we did not find any model that was developed and validated for this setting [1]. Trauma care providers in low and middle-income countries should have access to prognostic models derived in cohorts including patients from these populations.

Although prognostic models for trauma should be developed following methodological guidelines, the quality appraisal revealed several areas of improvement for most models. We found methodological limitations pertaining to issues such as inadequate methods to develop the prognostic models, handling of continuous variables and dealing with missing data. The MGAP was the one that fulfilled most of the recommended methodological quality items.

For a prognostic model to be used it should be well accepted by EMS providers. However, none of the studies evaluated the "acceptability" and "practicality" of the prognostic model. For a model to be effective it should improve patients' outcomes when tested in a randomised clinical trial, nevertheless the impact was not evaluated for any of the models. All models successfully discussed the rationale to include the predictors and included clearly defined predictors and valid outcomes.

We acknowledge that his systematic review has limitations. Some relevant studies may not have been located during our database search. Our literature review was only conducted in MEDLINE, although several other databases exist. The search strategy used in MEDLINE performed with high sensitivity (4 939 records retrieved) and low specificity (14 included studies). We identified three of the included studies through alternative sources (bibliographies); however, all 14 studies are included in MEDLINE. Closer examination of the included studies indicated inconsistent indexing of articles on prognostic scoring in adult trauma on MEDLINE. In the future, more homogenous reporting of studies pertaining to prognostic trauma models may reduce these limitations. Further, our exclusion of non-English language has contributed to the risk of missing relevant studies. However, we identified all the models included in a recently published triage guideline [39].

We only identified 9 validation studies indicating a need for further evaluation of performance transportability. In order to be able to evaluate the validity of future prognostic models we recommend to report the items included in our quality appraisal list (see additional file 3) as well as other relevant standards for reporting [40,41].

Our review should be incentives to further evolve the accuracy of prognostic models for the early care of trauma patients.

## Conclusions

This systematic review located and appraised the quality of five prognostic models for the early care of trauma patients. The prognostic models reported various outcomes pertaining to major trauma, but all models evaluated survival as an outcome. The general impression is that all models predict survival adequately. The MGAP fulfilled most of the suggested methodological quality items and is recommendable for routine use. However, there are many areas for improvement, including model development, analysis of continuous measures, handling of missing data, practicality and impact analysis.

## List of abbreviations used

ACS-COT: American College of Surgeons-Committee on Trauma; AUC: Area Under ROC Curve; CRAMS: Circulation, Respiration, Abdomen, Motor, Speech; ED: Emergency Department; EMS: Emergency Medical Service; GCS: Glasgow Coma Scale; ISS: Injury Severity Score; MGAP: Mechanism, GCS, Age, and Arterial Pressure; PHI: Pre-Hospital Index; PSS: Physiologic Severity Score; ROC: Receiver Operating Characteristics; RR: Respiratory Rate; SBP: Systolic Blood Pressure; TS: Trauma Score; T-RTS: Triage-Revised Trauma Score.

## Competing interests

The authors declare that they have no competing interests.

## Authors' contributions

MR, PP and HML developed the protocol. MR and PP conducted the systematic review. KB conducted the literature search. MR and PP conducted the data extraction. All authors read and approved the final manuscript.

## Supplementary Material

Additional file 1 **Literature search strategy**. Electronic bibliographical databases and search strategies Click here for file

Additional file 2 **Excluded studies**. List of full text studies excluded, with reason Click here for file

Additional file 3 **Quality assessment items list**. Items used to appraise quality of included prognostic model derivation studies Click here for file

Additional file 4 **Characteristics of investigated outcomes**. Table of outcomes pertaining to mortality, morbidity, process, anatomic injury and definition of "major trauma" Click here for file
