# Supplementary material for: Prognostic models for the early care of trauma patients: a systematic review
Source: Scand J Trauma Resusc Emerg Med. 2011 Mar 20;19:17. doi: 10.1186/1757-7241-19-17 (PMC3068084; doi:10.1186/1757-7241-19-17)
Supplement: Additional file 1 — Literature search strategy. Electronic bibliographical databases and search strategies [file 1757-7241-19-17-S1.DOC]

**Additional File 1; Search strategy:**

**MEDLINE search 1989 to May (week 1) 2010: 4880 records**

1. exp "Wounds and Injuries"/
2. exp abdominal injuries/
3. exp thoracic injuries/
4. exp spinal injuries/
5. exp spinal cord injuries/
6. exp spinal fractures/
7. exp rib fractures/
8. exp wounds, penetrating/
9. exp wounds, nonpenetrating/
10. exp wounds, stab/
11. exp wounds, gunshot/
12. exp multiple trauma/
13. exp shock, traumatic/
14. exp shock/
15. exp shock, hemorrhagic/
16. ((spin* or spinal cord or abdom* or chest or thora* or torso) adj3 (wound* or trauma* or injur* or oedema* or edema* or damag*)).ab,ti.
17. exp Crush Syndrome/
18. exp Hemorrhage/
19. exp triage/
20. or/1-19
21. *Trauma Severity Indices/
22. *Severity of Illness Index/
23. *Injury Severity Score/
24. *Prognosis/
25. *Risk Assessment/
26. *Risk Factors/
27. ((prognosis or predict* or risk*) adj3 (classif* or score* or assessment* or scale* or index or indices)).ab,ti.
28. (AIS or ISS).ti.
29. or/21-28
30. exp Humans/
31. (1989* or 1990* or 1991* or 1992* or 1993* or 1994* or 1995* or 1996* or 1997* or 1998* or 1999* or 2000* or 2001* or 2002* or 2003* or 2004* or 2005* or 2006* or 2008* or 2009* or 2010*).em.
32. 20 and 29 and 30 and 31
