# Supplementary material for: Prognostic models for the early care of trauma patients: a systematic review
Source: Scand J Trauma Resusc Emerg Med. 2011 Mar 20;19:17. doi: 10.1186/1757-7241-19-17 (PMC3068084; doi:10.1186/1757-7241-19-17)
Supplement: Additional file 2 — Excluded studies. List of full text studies excluded, with reason [file 1757-7241-19-17-S2.DOC]

**Additional File 2; Full text studies excluded, with reasons:**

| **Ref.** | **Author-year** | **Reason for exclusion** |
| --- | --- | --- |
| [1] | Alvarez-98 | Includes administrative data/data unavailable in the field, pertains only to TBI |
| [2] | Armagan-08 | ED-triage score |
| [3] | Bass-10 | Post-mortem dataset |
| [4] | Baxt-95 | Comment |
| [5] | Baxt-90 | Criteria only, no prognostic model |
| [6] | Benzer-91 | GCS like scale |
| [7] | Benzer-95 | GCS and GCS like scale |
| [8] | Bertollo-94 | Includes medical patients |
| [9] | Birkhahn-05 | Investigates healthy blood donors |
| [10] | Bouillon | Includes paediatric patients |
| [11] | Boyle-08 | Criteria only, no prognostic model |
| [12] | Brenneman-98 | Includes administrative data/data unavailable in the field |
| [13] | Burch-08 | Medical patients only |
| [14] | Burstein-96 | Investigates data quality |
| [15] | Cannon-09 | Investigates index, not a prognostic model |
| [16] | Champion-95 | Comment |
| [17] | Chesnut-98 | Comment |
| [18] | Cinelli-09 | Includes administrative data/data unavailable in the field |
| [19] | Clark-06 | Includes administrative data/data unavailable in the field |
| [20] | Clemmer-85 | Validation study published prior to 1989 |
| [21] | Cooke-06 | Includes para-clinical data and evaluates criteria but do not combine them in a prognostic model |
| [22] | Cooper-02 | Validates criteria, but do not combine them in a model |
| [23] | Cox-10 | Validates criteria, but do not combine them in a model |
| [24] | Croce-92 | Includes para-clinical data |
| [25] | Curet-00 | Does not combine predictive variables in a model |
| [26] | Dadley-98 | ED-triage score |
| [27] | Eckstein-03 | Evaluates GCS |
| [28] | Eichelberger-89 | Includes paediatric patients |
| [29] | Eitel-03 | ED-triage |
| [30] | Emerman-91 | Includes paediatric patients |
| [31] | Erickson-97 | Includes medical conditions |
| [32] | Esposito-95 | Validates criteria, but do not combine them in a model |
| [33] | Feldman-91 | Includes radiology and ICP |
| [34] | Fernandes-05 | Review article |
| [35] | Frankema-04 | Comment |
| [36] | Fries-94 | Validates criteria, but do not combine them in a model |
| [37] | Gabbe-03 | Review article |
| [38] | Gabbe-05 | Includes administrative data/data unavailable in the field |
| [39] | Gaddis-94 | Discuss statistical aspects of RTS/GCS |
| [40] | Gilboy-05 | Comment |
| [41] | Gill-06 | Evaluates GCS |
| [42] | Gill-05 | Pertains to TBI only |
| [43] | Gilpin-91 | Age NA |
| [44] | Goldhill-04 | Includes medical patients and use para–clinical data |
| [45] | Gottschalk | Propose a triage algorithm that include TEWS score, but present no data |
| [46] | Gray-97 | Includes paediatric patients |
| [47] | Hannan-05 | Criteria only, no prognostic model |
| [48] | Hannan-05 | Validates injury severity scores that requires data unavailable in the field. It combines these scores with physiological data |
| [49] | Hanson-94 | Comment |
| [50] | Healey-03 | Validates and statistically reappraise GCS |
| [51] | Hedges-87 | Validation study published in 1987 |
| [52] | Henry-06 | Validates criteria, but do not combine them in a model |
| [53] | Henry-96 | Validates criteria, but do not combine them in a model |
| [54] | Henry-96 | Validates criteria, but do not combine them in a model |
| [55] | Holcomb-05 | Validates criteria, but do not combine them in a model |
| [56] | Holmes-97 | Validates criteria, but do not combine them in a model |
| [57] | Johnstone-93 | Pertains to TBI only |
| [58] | Jones-89 | Validates criteria, but do not combine them in a model |
| [59] | Jones-95 | Validates criteria, but do not combine them in a model |
| [60] | Kennedy-93 | Pertains to TBI only |
| [61] | Kilgo-03 | Includes administrative data/data unavailable in the field |
| [62] | King-09 | Criteria only, no prognostic model, para-clinical data included |
| [63] | King-94 | Includes administrative data/data unavailable in the field |
| [64] | King-96 | Evaluates an index, not a prognostic model |
| [65] | Koehler-87 | Validates PHI, but published in 1987 |
| [66] | Kuhls-02 | Includes data unavailable in the field |
| [67] | Lubin-05 | Portrays triage precision and does not utilize a model |
| [68] | Luk-99 | Includes only patients with TS<5 |
| [69] | McLellan-89 | Includes para-clinical data |
| [70] | Meredith-02 | Includes administrative data/data unavailable in the field |
| [71] | Meredith-95 | Includes GCS only |
| [72] | Milham-04 | Includes administrative data/data unavailable in the field |
| [73] | Moore-06 | Includes GCS only |
| [74] | Moore-06 | Evaluates case-mix RTS only |
| [75] | Morris-90 | Includes administrative data/data unavailable in the field |
| [76] | Morris-90 | Includes administrative data/data unavailable in the field |
| [77] | Moussally-06 | Comment |
| [78] | Mulholland-08 | RTS validation is not main objective |
| [79] | Navin-09 | Validates a model where derivation study is ineligible |
| [80] | Norwood-02 | Validates GCS |
| [81] | Palanca-03 | Validates criteria, but do not combine them in a model |
| [82] | Pearl-08 | Includes data unavailable in the field |
| [83] | Phillips-93 | Validates criteria, but do not combine them in a model |
| [84] | Polin-95 | Pertains to TBI only |
| [85] | Purtil-08 | Validates criteria, but do not combine them in a model |
| [86] | Qureshi-04 | Comment |
| [87] | Rady-92 | Animal study |
| [88] | Rady-92 | Evaluates an index, not a prognostic model |
| [89] | Rady-94 | Includes medical patients |
| [90] | Raum-09 | Includes para-clinical data |
| [91] | Redmond-94 | Validates criteria, but do not combine them in a model |
| [92] | Rees-04 | Includes medical patients |
| [93] | Reisner-08 | Uses archived travel monitor data |
| [94] | Rhee-87 | Includes medical patients |
| [95] | Rhee-90 | Includes medical patients |
| [96] | Rocca-89 | Includes data unavailable in the field |
| [97] | Rodenberg-92 | Evaluate the effect of aircraft model on RTS |
| [98] | Rodenberg-96 | Comment |
| [99] | Ross-98 | Validates GCS |
| [100] | Rozycki-93 | Comment |
| [101] | Ruger-07 | Includes medical conditions |
| [102] | Sacco-93 | Includes administrative data/data unavailable in the field |
| [103] | Sacco-99 | Includes administrative data/data unavailable in the field |
| [104] | Sacco-05 | Derivation study on “RPM” is not accessible |
| [105] | Sacco-07 | Derivation study on “RPM” is not accessible |
| [106] | Sampalis-96 | Includes paediatric patients |
| [107] | Shaibani-90 | Includes administrative data/data unavailable in the field |
| [108] | Shanti-03 | Includes administrative data/data unavailable in the field |
| [109] | Shapiro-03 | Validates criteria, but do not combine them in a model |
| [110] | Shatney-94 | Validates criteria, but do not combine them in a model |
| [111] | Sikic-01 | Pertains to penetrating abdominal injuries only |
| [112] | Simmons-95 | Validates criteria, but do not combine them in a model |
| [113] | Simon-94 | Validates criteria, but do not combine them in a model |
| [114] | Smith-90 | Includes administrative data/data unavailable in the field |
| [115] | Smith-90 | Includes paediatric patients |
| [116] | Stoffel-09 | Pertains to penetrating abdominal injuries only |
| [117] | Subbe-06 | Combined medical and trauma patients |
| [118] | Taiwar-99 | Evaluates Trauma Score only |
| [119] | Tanabe-04 | Includes medical conditions |
| [120] | Teijink-93 | Includes administrative data/data unavailable in the field |
| [121] | Tinkoff-02 | Validates criteria, but do not combine them in a model |
| [122] | Tran-94 | Comment |
| [123] | Udekwu-04 | Pertains to TBI patients only |
| [124] | Wagner-00 | Pertains to TBI patients only |
| [125] | Yealy-94 | Comment |
| [126] | Zafonte-96 | Pertains to TBI patients only |
| [127] | Zafonte-96 | Pertains to TBI patients only |
| [128] | Zarzaur-08 | Evaluates an index not a model |
| [129] | Zechnich-95 | Validates criteria, but do not combine them in a model |
| Note:  TBI=Traumatic Brain Injury; GCS=Glasgow Coma Scale; NA=Not Available; ED=Emergency Department | | |

References

1. Alvarez M, Nava JM, Rue M, Quintana S: **Mortality prediction in head trauma patients: performance of Glasgow Coma Score and general severity systems.** *Crit Care Med* 1998, **26:**142-148.

2. Armagan E, Yilmaz Y, Olmez OF, Simsek G, Gul CB: **Predictive value of the modified Early Warning Score in a Turkish emergency department.** *European Journal of Emergency Medicine* 2008, **15:**338-340.

3. Bass CR, Salzar RS, Lucas SR, Rafaels KA, Damon AM, Crandall JR: **Re-evaluating the neck injury index (NII) using experimental PMHS tests.** *Traffic Injury Prevention* 2010, **11:**194-201.

4. Baxt WG: **The comparison of injury severity instrument performance using likelihood ration and ROC curve analyses.** *Journal of Trauma-Injury Infection & Critical Care* 1995, **39:**617-618.

5. Baxt WG, Jones G, Fortlage D: **The trauma triage rule: a new, resource-based approach to the prehospital identification of major trauma victims.** *Ann Emerg Med* 1990, **19:**1401-1406.

6. Benzer A, Mitterschiffthaler G, Marosi M, Luef G, Puhringer F, De La Renotiere K, Lehner H, Schmutzhard E: **Prediction of non-survival after trauma: Innsbruck Coma Scale.** *Lancet* 1991, **338:**977-978.

7. Benzer A, Traweger C, Ofner D, Marosi M, Luef G, Schmutzhard E: **Statistical modelling in analysis of outcome after trauma Glasgow-Coma-Scale and Innsbruck-Coma-Scale.** *Anasthesiologie, Intensivmedizin, Notfallmedizin, Schmerztherapie* 1995, **30:**231-235.

8. Bertollo S, Rodenberg H: **Correlation of the RTS (Revised Trauma Score) and RAPS (Rapid Acute Physiology Score) in rotor-wing prehospital care.** *Air Med J* 1994, **13:**91-95.

9. Birkhahn RH, Gaeta TJ, Terry D, Bove JJ, Tloczkowski J: **Shock index in diagnosing early acute hypovolemia.** *Am J Emerg Med* 2005, **23:**323-326.

10. Bouillon B, Lefering R, Vorweg M, Tiling T, Neugebauer E, Troidl H: **Trauma score systems: Cologne Validation Study.** *Journal of Trauma-Injury Infection & Critical Care* 1997, **42:**652-658.

11. Boyle MJ, Smith EC, Archer F: **Is mechanism of injury alone a useful predictor of major trauma?** *Injury* 2008, **39:**986-992.

12. Brenneman FD, Boulanger BR, McLellan BA, Redelmeier DA: **Measuring injury severity: time for a change?** *J Trauma* 1998, **44:**580-582.

13. Burch VC, Tarr G, Morroni C: **Modified early warning score predicts the need for hospital admission and inhospital mortality.** *Emergency Medicine Journal* 2008, **25:**674-678.

14. Burstein JL, Henry MC, Alicandro JM, McFadden K, Thode HC, Jr., Hollander JE: **Evidence for and impact of selective reporting of trauma triage mechanism criteria.** *Acad Emerg Med* 1996, **3:**1011-1015.

15. Cannon CM, Braxton CC, Kling-Smith M, Mahnken JD, Carlton E, Moncure M: **Utility of the shock index in predicting mortality in traumatically injured patients.** *Journal of Trauma-Injury Infection & Critical Care* 2009, **67:**1426-1430.

16. Champion HR, Sacco WJ, Copes WS: **Injury severity scoring again.** *Journal of Trauma-Injury Infection & Critical Care* 1995, **38:**94-95.

17. Chesnut RM: **Glasgow Coma Score versus severity systems in head trauma.** *Critical Care Medicine* 1998, **26:**10-11.

18. Cinelli SM, Brady P, Rennie CP, Tuluca C, Hall TS: **Comparative results of trauma scoring systems in fatal outcomes.** *Connecticut Medicine* 2009, **73:**261-265.

19. Clark DE, Ahmad S: **Estimating injury severity using the Barell matrix.** *Injury Prevention* 2006, **12:**111-116.

20. Clemmer TP, Orme JF, Jr., Thomas F, Brooks KA: **Prospective evaluation of the CRAMS scale for triaging major trauma.** *J Trauma* 1985, **25:**188-191.

21. Cooke WH, Salinas J, Convertino VA, Ludwig DA, Hinds D, Duke JH, Moore FA, Holcomb JB: **Heart rate variability and its association with mortality in prehospital trauma patients.** *J Trauma* 2006, **60:**363-370; discussion 370.

22. Cooper RJ, Schriger DL, Flaherty HL, Lin EJ, Hubbell KA: **Effect of vital signs on triage decisions.** *Ann Emerg Med* 2002, **39:**223-232.

23. Cox S, Smith K, Currell A, Harriss L, Barger B, Cameron P: **Differentiation of confirmed major trauma patients and potential major trauma patients using pre-hospital trauma triage criteria.** *Injury* 2010.

24. Croce MA, Fabian TC, Stewart RM, Pritchard FE, Minard G, Kudsk KA: **Correlation of abdominal trauma index and injury severity score with abdominal septic complications in penetrating and blunt trauma.** *Journal of Trauma-Injury Infection & Critical Care* 1992, **32:**380-387; discussion 387-388.

25. Curet MJ, Schermer CR, Demarest GB, Bieneik EJ, 3rd, Curet LB: **Predictors of outcome in trauma during pregnancy: identification of patients who can be monitored for less than 6 hours.** *Journal of Trauma-Injury Infection & Critical Care* 2000, **49:**18-24; discussion 24-15.

26. Dadley L: **Trauma scoring in A&E.** *Emergency Nurse* 1998, **6:**15-17.

27. Eckstein M: **The value of prehospital Glasgow Coma Scale (GCS) to predict the need for patient hospitalization.** *Journal of Trauma-Injury Infection & Critical Care* 2003, **54:**628; author reply 628-629.

28. Eichelberger MR, Gotschall CS, Sacco WJ, Bowman LM, Mangubat EA, Lowenstein AD: **A comparison of the trauma score, the revised trauma score, and the pediatric trauma score.** *Annals of Emergency Medicine* 1989, **18:**1053-1058.

29. Eitel DR, Travers DA, Rosenau AM, Gilboy N, Wuerz RC: **The emergency severity index triage algorithm version 2 is reliable and valid.** *Academic Emergency Medicine* 2003, **10:**1070-1080.

30. Emerman CL, Shade B, Kubincanek J: **A comparison of EMT judgment and prehospital trauma triage instruments.** *Journal of Trauma-Injury Infection & Critical Care* 1991, **31:**1369-1375.

31. Erickson TB, Koenigsberg M, Bunney EB, Schurgin B, Levy P, Willens J, Tanner L: **Prehospital severity scoring at major rock concert events.** *Prehospital & Disaster Medicine* 1997, **12:**195-199.

32. Esposito TJ, Offner PJ, Jurkovich GJ, Griffith J, Maier RV: **Do prehospital trauma center triage criteria identify major trauma victims?** *Arch Surg* 1995, **130:**171-176.

33. Feldman Z, Contant CF, Robertson CS, Narayan RK, Grossman RG: **Evaluation of the Leeds prognostic score for severe head injury.** *Lancet* 1991, **337:**1451-1453.

34. Fernandes CMB, Tanabe P, Gilboy N, Johnson LA, McNair RS, Rosenau AM, Sawchuk P, Thompson DA, Travers DA, Bonalumi N, Suter RE: **Five-level triage: a report from the ACEP/ENA Five-level Triage Task Force.** *Journal of Emergency Nursing* 2005, **31:**39-50; quiz 118.

35. Frankema SPG, Steyerberg EW, Harrell FE, Jr.: **The use of single or multiple injury descriptions in the assessment of injury severity.** *Journal of Trauma-Injury Infection & Critical Care* 2004, **56:**928; author reply 929.

36. Fries GR, McCalla G, Levitt MA, Cordova R: **A prospective comparison of paramedic judgment and the trauma triage rule in the prehospital setting.** *Ann Emerg Med* 1994, **24:**885-889.

37. Gabbe BJ, Cameron PA, Finch CF: **Is the revised trauma score still useful?** *ANZ Journal of Surgery* 2003, **73:**944-948.

38. Gabbe BJ, Magtengaard K, Hannaford AP, Cameron PA: **Is the Charlson Comorbidity Index useful for predicting trauma outcomes?** *Academic Emergency Medicine* 2005, **12:**318-321.

39. Gaddis GM, Gaddis ML: **Non-normality of distribution of Glasgow Coma Scores and Revised Trauma Scores.** *Ann Emerg Med* 1994, **23:**75-80.

40. Gilboy N, Tanabe P, Travers DA: **The Emergency Severity Index Version 4: changes to ESI level 1 and pediatric fever criteria.** *Journal of Emergency Nursing* 2005, **31:**357-362.

41. Gill M, Steele R, Windemuth R, Green SM: **A comparison of five simplified scales to the out-of-hospital Glasgow Coma Scale for the prediction of traumatic brain injury outcomes.** *Academic Emergency Medicine* 2006, **13:**968-973.

42. Gill M, Windemuth R, Steele R, Green SM: **A comparison of the Glasgow Coma Scale score to simplified alternative scores for the prediction of traumatic brain injury outcomes.** *Annals of Emergency Medicine* 2005, **45:**37-42.

43. Gilpin DA, Nelson PG: **Revised trauma score: a triage tool in the accident and emergency department.** *Injury* 1991, **22:**35-37.

44. Goldhill DR, McNarry AF: **Physiological abnormalities in early warning scores are related to mortality in adult inpatients.** *Br J Anaesth* 2004, **92:**882-884.

45. Gottschalk SB, Wood D, DeVries S, Wallis LA, Bruijns S: **The Cape Triage Score: a new triage system South Africa. Proposal from the Cape Triage Group.** *Emerg Med J* 2006, **23:**149-153.

46. Gray A, Goyder EC, Goodacre SW, Johnson GS: **Trauma triage: a comparison of CRAMS and TRTS in a UK population.** *Injury* 1997, **28:**97-101.

47. Hannan EL, Farrell LS, Cooper A, Henry M, Simon B, Simon R: **Physiologic trauma triage criteria in adult trauma patients: are they effective in saving lives by transporting patients to trauma centers?** *J Am Coll Surg* 2005, **200:**584-592.

48. Hannan EL, Waller CH, Farrell LS, Cayten CG: **A comparison among the abilities of various injury severity measures to predict mortality with and without accompanying physiologic information.** *Journal of Trauma-Injury Infection & Critical Care* 2005, **58:**244-251.

49. Hanson WB: **Predicting severity of trauma.** *Southern Medical Journal* 1994, **87:**419.

50. Healey C, Osler TM, Rogers FB, Healey MA, Glance LG, Kilgo PD, Shackford SR, Meredith JW: **Improving the Glasgow Coma Scale score: motor score alone is a better predictor.** *J Trauma* 2003, **54:**671-678; discussion 678-680.

51. Hedges JR, Feero S, Moore B, Haver DW, Shultz B: **Comparison of prehospital trauma triage instruments in a semirural population.** *J Emerg Med* 1987, **5:**197-208.

52. Henry MC: **Trauma triage: New York experience.** *Prehosp Emerg Care* 2006, **10:**295-302.

53. Henry MC, Alicandro JM, Hollander JE, Moldashel JG, Cassara G, Thode HC, Jr.: **Evaluation of American College of Surgeons trauma triage criteria in a suburban and rural setting.** *Am J Emerg Med* 1996, **14:**124-129.

54. Henry MC, Hollander JE, Alicandro JM, Cassara G, O'Malley S, Thode HC, Jr.: **Incremental benefit of individual American College of Surgeons trauma triage criteria.** *Academic Emergency Medicine* 1996, **3:**992-1000.

55. Holcomb JB, Niles SE, Miller CC, Hinds D, Duke JH, Moore FA: **Prehospital physiologic data and lifesaving interventions in trauma patients.** *Mil Med* 2005, **170:**7-13.

56. Holmes JF, Baier ME, Derlet RW: **Failure of the Miller criteria to predict significant intracranial injury in patients with a Glasgow Coma Scale score of 14 after minor head trauma.** *Academic Emergency Medicine* 1997, **4:**788-792.

57. Johnstone AJ, Lohlun JC, Miller JD, McIntosh CA, Gregori A, Brown R, Jones PA, Anderson SI, Tocher JL: **A comparison of the Glasgow Coma Scale and the Swedish Reaction Level Scale.** *Brain Injury* 1993, **7:**501-506.

58. Jones IS, Champion HR: **Trauma triage: vehicle damage as an estimate of injury severity.** *J Trauma* 1989, **29:**646-653.

59. Jones JM, Maryosh J, Johnstone S, Templeton J: **A multivariate analysis of factors related to the mortality of blunt trauma admissions to the North Staffordshire Hospital Centre.** *J Trauma* 1995, **38:**118-122.

60. Kennedy F, Gonzalez P, Dang C, Fleming A, Sterling-Scott R: **The Glasgow Coma Scale and prognosis in gunshot wounds to the brain.** *Journal of Trauma-Injury Infection & Critical Care* 1993, **35:**75-77.

61. Kilgo PD, Osler TM, Meredith W: **The worst injury predicts mortality outcome the best: rethinking the role of multiple injuries in trauma outcome scoring.** *J Trauma* 2003, **55:**599-606; discussion 606-597.

62. King DR, Ogilvie MP, Pereira BM, Chang Y, Manning RJ, Conner JA, Schulman CI, McKenney MG, Proctor KG: **Heart rate variability as a triage tool in patients with trauma during prehospital helicopter transport.** *J Trauma* 2009, **67:**436-440.

63. King PM, Tucker WS, Waddell JP, Brown T: **Correlation of trauma scoring and outcome in a Canadian trauma centre.** *Canadian Journal of Surgery* 1994, **37:**185-188.

64. King RW, Plewa MC, Buderer NM, Knotts FB: **Shock index as a marker for significant injury in trauma patients.** *Acad Emerg Med* 1996, **3:**1041-1045.

65. Koehler JJ, Malafa SA, Hillesland J, Baer LJ, Rogers RN, Navitskas NR, Briggs D, Simpson D, Roller B, Lilleboe P, et al.: **A multicenter validation of the prehospital index.** *Ann Emerg Med* 1987, **16:**380-385.

66. Kuhls DA, Malone DL, McCarter RJ, Napolitano LM: **Predictors of mortality in adult trauma patients: the physiologic trauma score is equivalent to the Trauma and Injury Severity Score.** *J Am Coll Surg* 2002, **194:**695-704.

67. Lubin JS, Delbridge TR, Cole JS, Nicholas DH, Fore CA, Wadas RJ: **EMS and emergency department physician triage: injury severity in trauma patients transported by helicopter.** *Prehospital Emergency Care* 2005, **9:**198-202.

68. Luk SS, Jacobs L, Ciraulo DL, Cortes V, Sable A, Cowell VL: **Outcome assessment of physiologic and clinical predictors of survival in patients after traumatic injury with a trauma score less than 5.** *J Trauma* 1999, **46:**122-128.

69. McLellan BA, Koch JP, Wortzman D, Rogers C, Szalai J, Williams D: **Early identification of high-risk patients using the "estimated" injury severity score and age.** *Accident Analysis & Prevention* 1989, **21:**283-290.

70. Meredith JW, Evans G, Kilgo PD, MacKenzie E, Osler T, McGwin G, Cohn S, Esposito T, Gennarelli T, Hawkins M, et al: **A comparison of the abilities of nine scoring algorithms in predicting mortality.** *Journal of Trauma-Injury Infection & Critical Care* 2002, **53:**621-628; discussion 628-629.

71. Meredith W, Rutledge R, Hansen AR, Oller DW, Thomason M, Cunningham P, Baker CC: **Field triage of trauma patients based upon the ability to follow commands: a study in 29,573 injured patients.** *J Trauma* 1995, **38:**129-135.

72. Millham FH, LaMorte WW: **Factors associated with mortality in trauma: re-evaluation of the TRISS method using the National Trauma Data Bank.** *Journal of Trauma-Injury Infection & Critical Care* 2004, **56:**1090-1096.

73. Moore L, Lavoie A, Camden S, Le Sage N, Sampalis JS, Bergeron E, Abdous B: **Statistical validation of the Glasgow Coma Score.** *J Trauma* 2006, **60:**1238-1243; discussion 1243-1234.

74. Moore L, Lavoie A, LeSage N, Abdous B, Bergeron E, Liberman M, Emond M: **Statistical validation of the Revised Trauma Score.** *J Trauma* 2006, **60:**305-311.

75. Morris JA, Jr., MacKenzie EJ, Damiano AM, Bass SM: **Mortality in trauma patients: the interaction between host factors and severity.** *J Trauma* 1990, **30:**1476-1482.

76. Morris JA, Jr., MacKenzie EJ, Edelstein SL: **The effect of preexisting conditions on mortality in trauma patients.** *JAMA* 1990, **263:**1942-1946.

77. Moussally J, Stair TO, Gilboy N, Lucenti MJ, Pallin DJ: **Characteristics of patients classified differently by versions 2 and 3 of the Emergency Severity Index.** *Journal of Emergency Medicine* 2006, **31:**229.

78. Mulholland SA, Cameron PA, Gabbe BJ, Williamson OD, Young K, Smith KL, Bernard SA: **Prehospital prediction of the severity of blunt anatomic injury.** *Journal of Trauma-Injury Infection & Critical Care* 2008, **64:**754-760.

79. Navin DM, Sacco WJ, McGill G: **Application of a new resource-constrained triage method to military-age victims.** *Military Medicine* 2009, **174:**1247-1255.

80. Norwood SH, McAuley CE, Berne JD, Vallina VL, Creath RG, McLarty J: **A prehospital glasgow coma scale score < or = 14 accurately predicts the need for full trauma team activation and patient hospitalization after motor vehicle collisions.** *J Trauma* 2002, **53:**503-507.

81. Palanca S, Taylor DM, Bailey M, Cameron PA: **Mechanisms of motor vehicle accidents that predict major injury.** *Emerg Med (Fremantle)* 2003, **15:**423-428.

82. Pearl A, Bar-Or R, Bar-Or D: **An artificial neural network derived trauma outcome prediction score as an aid to triage for non-clinicians.** *Studies in Health Technology & Informatics* 2008, **136:**253-258.

83. Phillips JA, Buchman TG: **Optimizing prehospital triage criteria for trauma team alerts.** *J Trauma* 1993, **34:**127-132.

84. Polin RS, Shaffrey ME, Phillips CD, Germanson T, Jane JA: **Multivariate analysis and prediction of outcome following penetrating head injury.** *Neurosurgery Clinics of North America* 1995, **6:**689-699.

85. Purtill M-A, Benedict K, Hernandez-Boussard T, Brundage SI, Kritayakirana K, Sherck JP, Garland A, Spain DA: **Validation of a prehospital trauma triage tool: a 10-year perspective.** *Journal of Trauma-Injury Infection & Critical Care* 2008, **65:**1253-1257.

86. Qureshi AH: **Evaluation of revised trauma score in polytraumatized patients.** *Jcpsp, Journal of the College of Physicians & Surgeons - Pakistan* 2004, **14:**644; author reply 644.

87. Rady MY, Nightingale P, Little RA, Edwards JD: **Shock index: a re-evaluation in acute circulatory failure.** *Resuscitation* 1992, **23:**227-234.

88. Rady MY, Rivers EP, Martin GB, Smithline H, Appelton T, Nowak RM: **Continuous central venous oximetry and shock index in the emergency department: use in the evaluation of clinical shock.** *Am J Emerg Med* 1992, **10:**538-541.

89. Rady MY, Smithline HA, Blake H, Nowak R, Rivers E: **A comparison of the shock index and conventional vital signs to identify acute, critical illness in the emergency department.[Erratum appears in Ann Emerg Med 1994 Dec;24(6):1208].** *Annals of Emergency Medicine* 1994, **24:**685-690.

90. Raum MR, Nijsten MWN, Vogelzang M, Schuring F, Lefering R, Bouillon B, Rixen D, Neugebauer EAM, Ten Duis HJ, Polytrauma Study Group of the German Trauma S: **Emergency trauma score: an instrument for early estimation of trauma severity.** *Critical Care Medicine* 2009, **37:**1972-1977.

91. Redmond AD, Redmond CA, Jones JM, Hillier V: **The significance of patient appearance in predicting severity of injury.** *Injury* 1994, **25:**81-82.

92. Rees JE, Mann C: **Use of the patient at risk scores in the emergency department: a preliminary study.** *Emergency Medicine Journal* 2004, **21:**698-699.

93. Reisner AT, Chen L, McKenna TM, Reifman J: **Automatically-computed prehospital severity scores are equivalent to scores based on medic documentation.** *Journal of Trauma-Injury Infection & Critical Care* 2008, **65:**915-923.

94. Rhee KJ, Fisher CJ, Jr., Willitis NH: **The Rapid Acute Physiology Score.** *Am J Emerg Med* 1987, **5:**278-282.

95. Rhee KJ, Mackenzie JR, Burney RE, Willits NH, O'Malley RJ, Reid N, Schwabe D, Storer DL, Weber R: **Rapid acute physiology scoring in transport systems.** *Crit Care Med* 1990, **18:**1119-1123.

96. Rocca B, Martin C, Viviand X, Bidet PF, Saint-Gilles HL, Chevalier A: **Comparison of four severity scores in patients with head trauma.** *J Trauma* 1989, **29:**299-305.

97. Rodenberg H: **Effect of aeromedical aircraft on care of trauma patients: evaluation using the Revised Trauma Score.** *Southern Medical Journal* 1992, **85:**1065-1071.

98. Rodenberg H: **Scoring systems in air medical transport: a primer.** *Air Medical Journal* 1996, **15:**184-190.

99. Ross SE, Leipold C, Terregino C, O'Malley KF: **Efficacy of the motor component of the Glasgow Coma Scale in trauma triage.** *J Trauma* 1998, **45:**42-44.

100. Rozycki G: **Trauma during pregnancy: predicting pregnancy outcome.** *Arch Gynecol Obstet* 1993, **253 Suppl:**S15-20.

101. Ruger JP, Lewis LM, Richter CJ: **Identifying high-risk patients for triage and resource allocation in the ED.** *Am J Emerg Med* 2007, **25:**794-798.

102. Sacco WJ, Copes WS, Bain LW, Jr., MacKenzie EJ, Frey CF, Hoyt DB, Weigelt JA, Champion HR: **Effect of preinjury illness on trauma patient survival outcome.** *Journal of Trauma-Injury Infection & Critical Care* 1993, **35:**538-542; discussion 542-533.

103. Sacco WJ, MacKenzie EJ, Champion HR, Davis EG, Buckman RF: **Comparison of alternative methods for assessing injury severity based on anatomic descriptors.** *J Trauma* 1999, **47:**441-446; discussion 446-447.

104. Sacco WJ, Navin DM, Fiedler KE, Waddell RK, 2nd, Long WB, Buckman RF, Jr.: **Precise formulation and evidence-based application of resource-constrained triage.** *Acad Emerg Med* 2005, **12:**759-770.

105. Sacco WJ, Navin DM, Waddell RK, 2nd, Fiedler KE, Long WB, Buckman RF, Jr.: **A new resource-constrained triage method applied to victims of penetrating injury.** *J Trauma* 2007, **63:**316-325.

106. Sampalis JS, Tamim H, Nikolis A, Lavoie A, Williams JI: **Predictive validity and internal consistency of the pre-hospital index measured on-site by physicians.** *Accident Analysis & Prevention* 1996, **28:**675-684.

107. Shaibani SJ, Baum HM: **Comparisons of the thoracic trauma index with other models.** *Accident Analysis & Prevention* 1990, **22:**35-45.

108. Shanti CM, Tyburski JG, Rishell KB, Wilson RF, Lozen Y, Seibert C, Steffes C, Carlin AM: **Correlation of revised trauma score and injury severity score (TRISS) predicted probability of survival with peer-reviewed determination of trauma deaths.** *American Surgeon* 2003, **69:**257-260; discussion 260.

109. Shapiro NI, Kociszewski C, Harrison T, Chang Y, Wedel SK, Thomas SH: **Isolated prehospital hypotension after traumatic injuries: a predictor of mortality?** *J Emerg Med* 2003, **25:**175-179.

110. Shatney CH, Sensaki K: **Trauma team activation for 'mechanism of injury' blunt trauma victims: time for a change?** *J Trauma* 1994, **37:**275-281; discussion 281-272.

111. Sikic N, Korac Z, Krajacic I, Zunic J: **War abdominal trauma: usefulness of Penetrating Abdominal Trauma Index, Injury Severity Score, and number of injured abdominal organs as predictive factors.** *Military Medicine* 2001, **166:**226-230.

112. Simmons E, Hedges JR, Irwin L, Maassberg W, Kirkwood HA, Jr.: **Paramedic injury severity perception can aid trauma triage.** *Annals of Emergency Medicine* 1995, **26:**461-468.

113. Simon BJ, Legere P, Emhoff T, Fiallo VM, Garb J: **Vehicular trauma triage by mechanism: avoidance of the unproductive evaluation.** *J Trauma* 1994, **37:**645-649.

114. Smith DP, Enderson BL, Maull KI: **Trauma in the elderly: determinants of outcome.** *Southern Medical Journal* 1990, **83:**171-177.

115. Smith JS, Jr., Bartholomew MJ: **Trauma index revisited: a better triage tool.** *Critical Care Medicine* 1990, **18:**174-180.

116. Stoffel M, Huser N, Kayser K, Kriner M, Degiannis E, Doll D: **Cerebral gunshot wounds: a score based on three clinical parameters to predict the risk of early mortality.** *ANZ Journal of Surgery* 2009, **79:**789-793.

117. Subbe CP, Slater A, Menon D, Gemmell L: **Validation of physiological scoring systems in the accident and emergency department.** *Emergency Medicine Journal* 2006, **23:**841-845.

118. Talwar S, Jain S, Porwal R, Laddha BL, Prasad P: **Trauma scoring in a developing country.** *Singapore Medical Journal* 1999, **40:**386-388.

119. Tanabe P, Gimbel R, Yarnold PR, Kyriacou DN, Adams JG: **Reliability and validity of scores on The Emergency Severity Index version 3.** *Acad Emerg Med* 2004, **11:**59-65.

120. Teijink JA, Dwars BJ, Patka P, Haarman HJ: **Scoring multitrauma patients: which scoring system?** *Injury* 1993, **24:**13-16.

121. Tinkoff GH, O'Connor RE: **Validation of new trauma triage rules for trauma attending response to the emergency department.** *J Trauma* 2002, **52:**1153-1158; discussion 1158-1159.

122. Tran DD: **Scoring systems for prediction of mortality in critical trauma.** *American Journal of Surgery* 1994, **167:**622-623.

123. Udekwu P, Kromhout-Schiro S, Vaslef S, Baker C, Oller D: **Glasgow Coma Scale score, mortality, and functional outcome in head-injured patients.** *Journal of Trauma-Injury Infection & Critical Care* 2004, **56:**1084-1089.

124. Wagner AK, Hammond FM, Grigsby JH, Norton HJ: **The value of trauma scores: predicting discharge after traumatic brain injury.** *American Journal of Physical Medicine & Rehabilitation* 2000, **79:**235-242.

125. Yealy DM, Delbridge TR: **The shock index: all that glitters.** *Annals of Emergency Medicine* 1994, **24:**714-715.

126. Zafonte RD, Hammond FM, Mann NR, Wood DL, Black KL, Millis SR: **Relationship between Glasgow coma scale and functional outcome.** *Am J Phys Med Rehabil* 1996, **75:**364-369.

127. Zafonte RD, Hammond FM, Mann NR, Wood DL, Millis SR, Black KL: **Revised trauma score: an additive predictor of disability following traumatic brain injury?** *American Journal of Physical Medicine & Rehabilitation* 1996, **75:**456-461.

128. Zarzaur BL, Croce MA, Fischer PE, Magnotti LJ, Fabian TC: **New vitals after injury: shock index for the young and age x shock index for the old.** *Journal of Surgical Research* 2008, **147:**229-236.

129. Zechnich AD, Hedges JR, Spackman K, Jui J, Mullins RJ: **Applying the trauma triage rule to blunt trauma patients.** *Acad Emerg Med* 1995, **2:**1043-1052.
