# Supplementary material for: Prognostic models for the early care of trauma patients: a systematic review
Source: Scand J Trauma Resusc Emerg Med. 2011 Mar 20;19:17. doi: 10.1186/1757-7241-19-17 (PMC3068084; doi:10.1186/1757-7241-19-17)
Supplement: Additional file 3 — Quality assessment items list. Items used to appraise quality of included prognostic model derivation studies [file 1757-7241-19-17-S3.DOC]

**Additional File 3; Quality appraisal list:**

**Internal validity**

**1) Did the patients have an adequate follow-up?**

All prognostic model studies are conceptually cohort–design, although the method of data collection could be either prospective or retrospective (i.e. use of databases). In cohort studies a large loss to follow-up could lead to attrition bias. To minimize attrition bias the follow-up should be at least 90% of the original cohort.

**2) Was a discussion included about rationale to include the predictors?**

The variables included should be important predictors reported in previous studies (i.e. respiratory rate or systolic blood pressure) or include a rationale for the variables included.

**3) Were the predictive variables clearly defined?**

Variable definition and measurement should be clearly described in the method section of the report.

**4) Were the outcomes predicted valid?**

Validity, for outcomes other than mortality, should be reported.

**5) Were missing data adequately managed?**

Imputation strategies are preferable to complete case analysis when the amount of missing data is large.

**6) Was an adequate strategy performed to build the multivariable model?**

Multivariable analysis strategy should consider clinical criteria when entering variables in the model and not only automatic selection strategies such as stepwise. In some cases important clinical predictors could be “forced” into the model.

**7) Were interactions between the variables examined?**

When a multivariable analysis is performed interactions between variables should be explored.

**8) Were continuous variables handled appropriately?**

It is preferable to keep continuous variables as originally recorded because they can give more information and are more powerful to detect an association. Categorization of a variable into groups assumes a constant risk in each group created, which is often not true. In the case where variables are categorized, the rationale for the cut-point should be clearly explained.

**9) Were more than 10 events per variable included?**

The estimates may be unreliable if the data contain less than 10 outcome events relative to the number of parameters.

**External validity**

**10) Was the description of the sample reported?**

To interpret generalizability it is important to know the characteristics of the sample from which the model was derived, studies should thus include information about the study population: e.g. time of inclusion of the patients in relation to the injury, time of the measurement of the variables, treatment received.

**11) Was it clearly explained how to estimate the prognosis?**

For a prognostic model to be clinically useful it should be clearly explained how to estimate the prognosis in a clinical setting. Probability of the outcome could be obtained through simple scores, nomograms, or simple figures. Reporting just the coefficients of the multivariable model is not enough to be clinically practical in the emergency setting.

**12) Were measures of accuracy reported?**

To evaluate a prognostic model’s performance, its discrimination and calibration should be assessed: Discrimination refers to the ability to rank in the correct order individuals with different prognosis. Calibration refers to the ability to predict correctly the prognosis (not too high or too low).

**13) Were confidence intervals presented?**

Clinicians that will use the prognostic model should know the precision of the estimates derived from the model.

**14) Was the prognostic model internally validated?**

Internal validation assesses the validity of the prognostic model for the setting where the development data originated.

**15) How many studies validated the prognostic model externally?**

For a prognostic model to be generalized to a population different from the one from which the model was derived, it should be evaluated (validated) in a different population (e.g. different geographical region, historical periods or different methods of data collection)

**16) Was the clinical credibility of the prognostic model evaluated?**

For a prognostic model to be used it should be well accepted by physicians. Ideally the “acceptability” and “practicality” of a prognostic model should be evaluated.

**17) Does the prognostic model improve clinical outcomes when tested in a randomised clinical trial?**

For a model to be effective it should improve patients’ outcomes when tested in a randomised clinical trial.
