# Supplementary material for: Prognostic models for the early care of trauma patients: a systematic review
Source: Scand J Trauma Resusc Emerg Med. 2011 Mar 20;19:17. doi: 10.1186/1757-7241-19-17 (PMC3068084; doi:10.1186/1757-7241-19-17)
Supplement: Additional file 4 — Characteristics of investigated outcomes. Table of outcomes pertaining to mortality, morbidity, process, anatomic injury and definition of "major trauma" [file 1757-7241-19-17-S4.DOC]

**Additional File 4; Characteristics of investigated outcomes**

| **Study** | **Outcomes**  Mortality | Morbidity | Process | Anatomic injury | Definition of “major trauma” |
| --- | --- | --- | --- | --- | --- |
| **CRAMS**  **Gormican-82** | Survival (ED) | NA | General/neuro/other surgery |  | Died in the ED; General/neuro surgery |
| Baxt-89 | Survival | Outcome scale  (5 levels) | NA | ISS≥10; ISS≥15; ISS≥20 | ISS≥15; Death or deficit |
| Emerman-92 | Survival | NA | General/neuro surgery<2 hrs | NA | Death; General/neuro surgery<2hrs |
| **PHI**  **Koehler-86** | Survival (72 hrs) | NA | General/neuro surgery<24 hrs | NA | General/neuro surgery<24 hrs; Death<72 hrs |
| Baxt-89 | Survival | Outcome scale  (5 levels) | NA | ISS≥10; ISS≥15; ISS≥20 | ISS≥15; Death or deficit |
| Emerman-92 | Survival | NA | General/neuro surgery <2 hrs | NA | Death; General/neuro surgery<2 hrs |
| Plant-95 | Survival (72 hrs) | NA | General/neuro surgery<4 hrs | ISS | NA |
| Bond-97 | NA | NA | NA | ISS>15 | ISS>15 |
| Tamim-02 | Survival (7 days) |  | Hospital LOS; Non-orthopaedic/non-plastic surgery (4days); ICU admission (7 days) | ISS | Non-orthopaedic/non-plastic surgery (4days); ICU admission (7 days); Death (7 days) |
| **T–RTS**  **Champion-89** | Survival | NA | NA | ISS | ISS>15 |
| Baxt-89 | Survival | Outcome scale  (5 levels) | NA | ISS≥10; ISS≥15; ISS≥20 | ISS≥15; Death or deficit |
| Emerman-92 | Survival | NA | General/neuro surgery <2 hrs | NA | Death; General/neuro surgery<2 hrs |
| Roorda-96 | Survival (48 hrs) | NA | Emergency surgery; ICU admittance | HTI-ISS≥18; HTI-ISS≥20; modified HTI-ISS criterion | HTI-ISS≥18 |
| Ahmad-04 | Survival | Disabilities  (Discharge) | Hospital LOS | NA | NA |
| Al-Salamah-04 | Survival (Hospital discharge) | GOS | ICU admission; Required intubation in the ED | NA | NA |
| Moore-06 | Survival | NA | Transfer | ISS≥12; Critical head injury* | NA |
| Sartorius-10 | Survival (30 days) | NA | NA | NA | Survival (30 days) |
| **PSS**  **Husum-03** | Survival | NA | NA | ISS>15 | ISS>15 |
| **MGAP**  **Sartorius-10** | Survival (30 days) | NA | NA | NA | Death (30 days) |
| *) at least one Abreviated Injury Scale (AIS) 5 or two AIS 4 of the head or neck;  NA=Not Available; ISS=Injury Severity Score; HTI=Hospital Trauma Index; hrs=hours; GOS=Glasgow Outcome Scale; ED=Emergency Department; CRAMS=Circulation, Respiration, Abdomen, Motor, Speech; PHI=Pre-Hospital Index; T-RTS=Triage-Revised Trauma Score; PSS=Physiologic Severity Score; MGAP=Mechanism, Glasgow Coma Scale, Age, and Arterial Pressure | | | | | |
